# Supplementary material for: Tera-MIND: Tera-scale mouse brain simulation via spatial mRNA-guided diffusion
Source: iScience. 2026 Jul 8;29(7):116355. doi: 10.1016/j.isci.2026.116355 (PMC13378011; doi:10.1016/j.isci.2026.116355)
Supplement: Document S1. Figures S1–S5 [file mmc1.pdf]

iScience, Volume *29*

Supplemental information

Tera-MIND: Tera-scale mouse brain simulation via spatial mRNA-guided diffusion

Jiqing Wu, Ingrid Berg, Yawei Li, Ender Konukoglu, and Viktor H. Koelzer

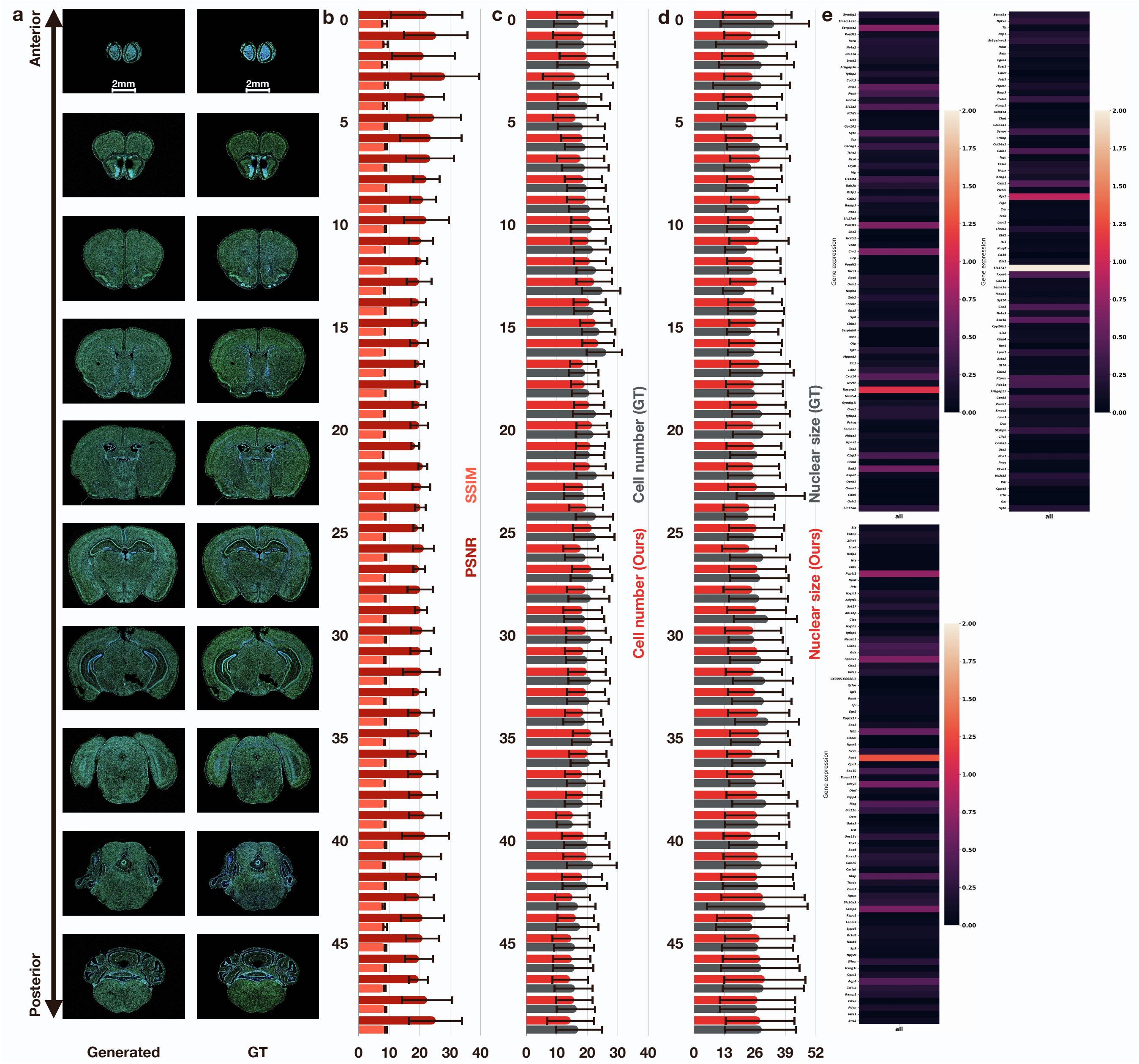

Figure 1: **The comprehensive generation, quantification, and gene expression statistics for the mouse brain reported as the Main result**. **a**. The side-by-side comparison between generated and GT WSIs. Here, we report the first of five consecutive slices for the sake of a clear visualization. **a**. For all the slices, we report the average PSNR and SSIM scores with standard deviation (std). **c**. For all the slices, we report the average cell number with std between our generated results and GT. **d**. For all the slices, we report the average nuclear size with std between our generated results and GT. **e**. The heatmap of overall 229-plex gene expression values normalized by $\log 2$. In **b**-**d**, data are presented as mean + standard deviation.

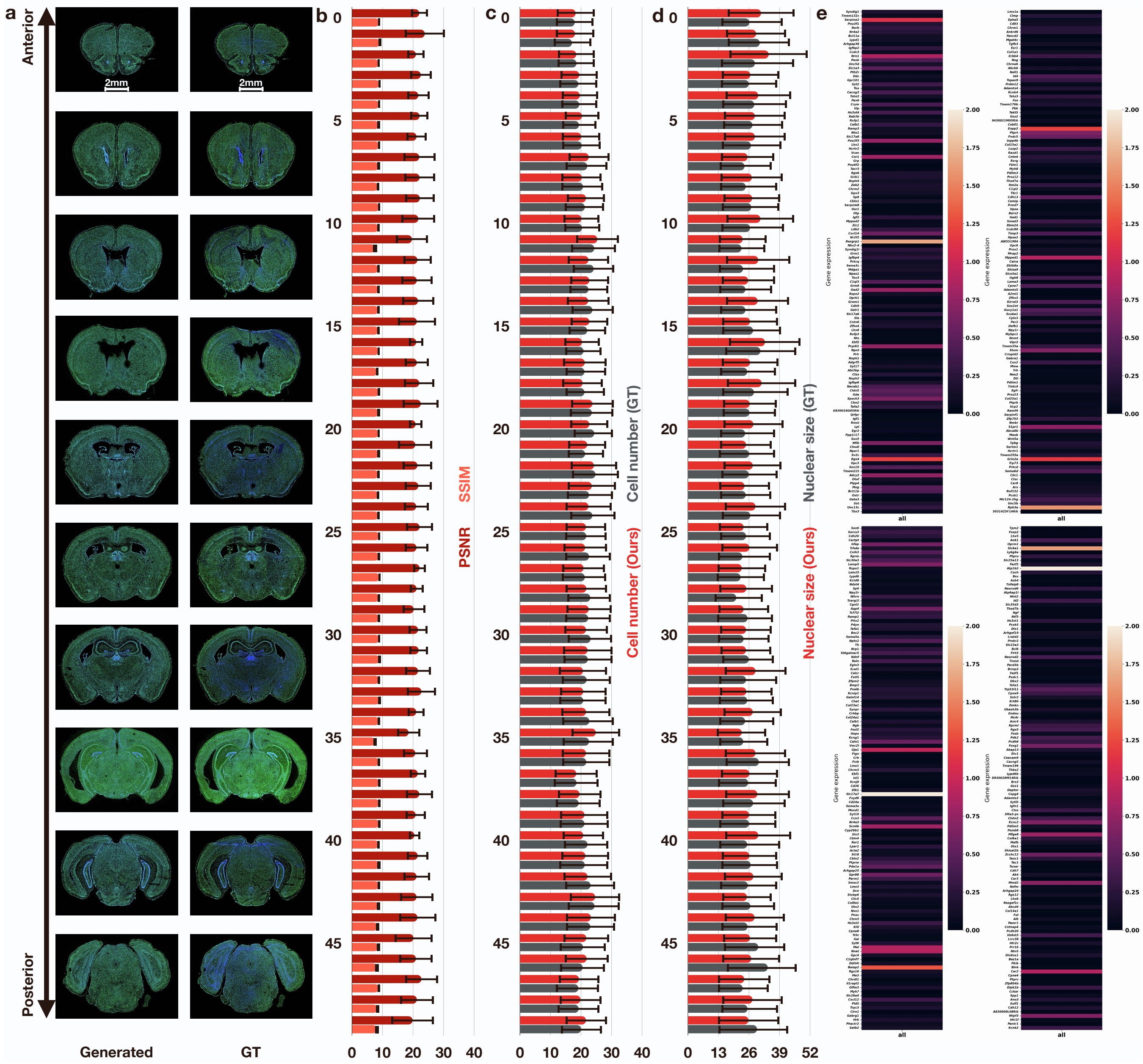

Figure 2: **The comprehensive generation, quantification, and gene expression statistics for the mouse brain reported as the Supp (m) result**. **a**. The side-by-side comparison between generated and GT WSIs. Here, we report the first of five consecutive slices for the sake of a clear visualization. **a**. For all the slices, we report the average PSNR and SSIM scores with standard deviation (std). **c**. For all the slices, we report the average cell number with std between our generated results and GT. **d**. For all the slices, we report the average nuclear size with std between our generated results and GT. **e**. The heatmap of overall 500-plex gene expression values normalized by $\log 2$. In **b**-**d**, data are presented as mean + standard deviation.

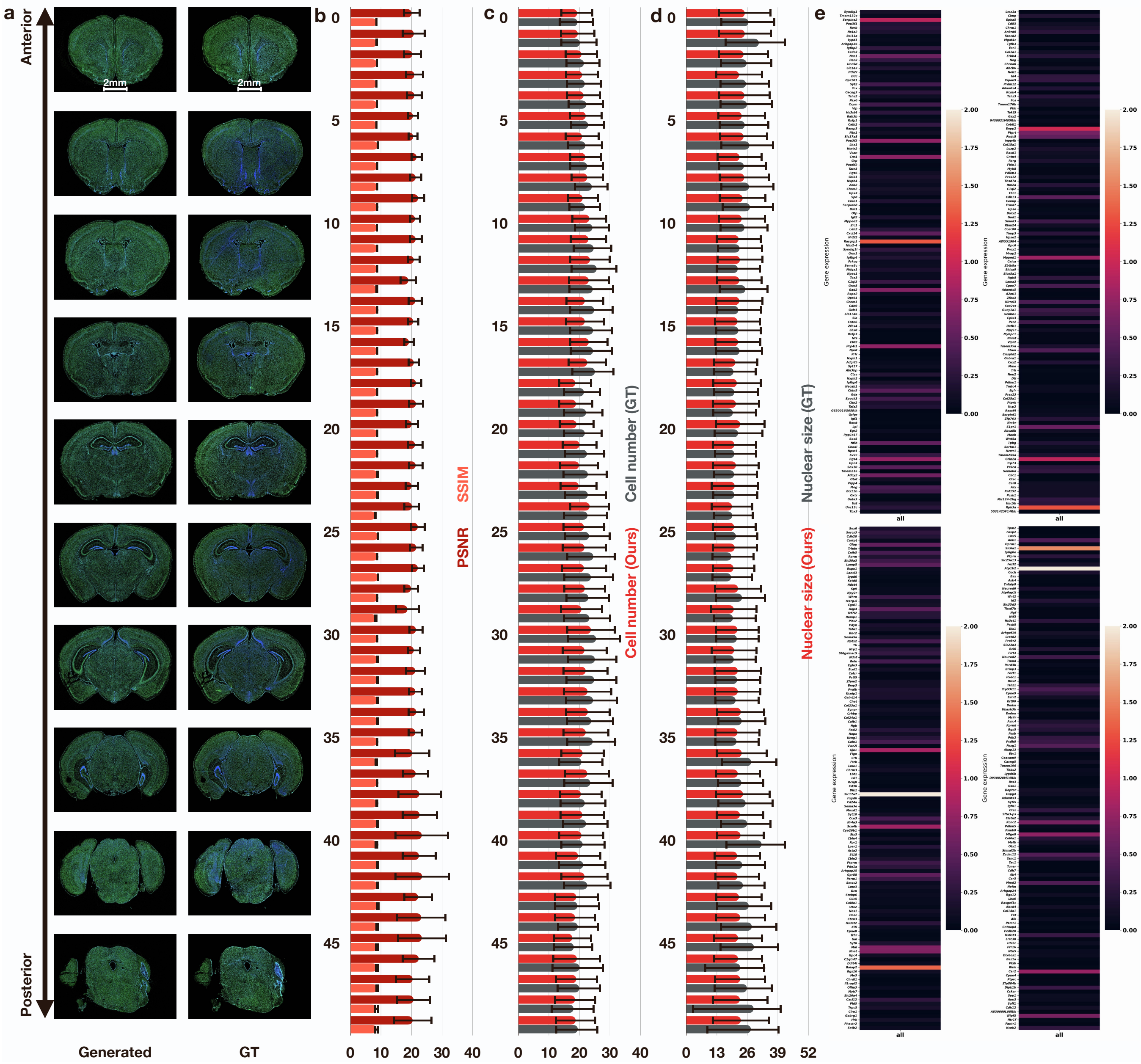

Figure 3: **The comprehensive generation, quantification, and gene expression statistics for the mouse brain reported as the Supp (f) result.** **a**. The side-by-side comparison between generated and GT WSIs. Here, we report the first of five consecutive slices for the sake of a clear visualization. **a**. For all the slices, we report the average PSNR and SSIM scores with standard deviation (std). **c**. For all the slices, we report the average cell number with std between our generated results and GT. **d**. For all the slices, we report the average nuclear size with std between our generated results and GT. **e**. The heatmap of overall 500-plex gene expression values normalized by $\log 2$. In **b**-**d**, data are presented as mean + standard deviation.

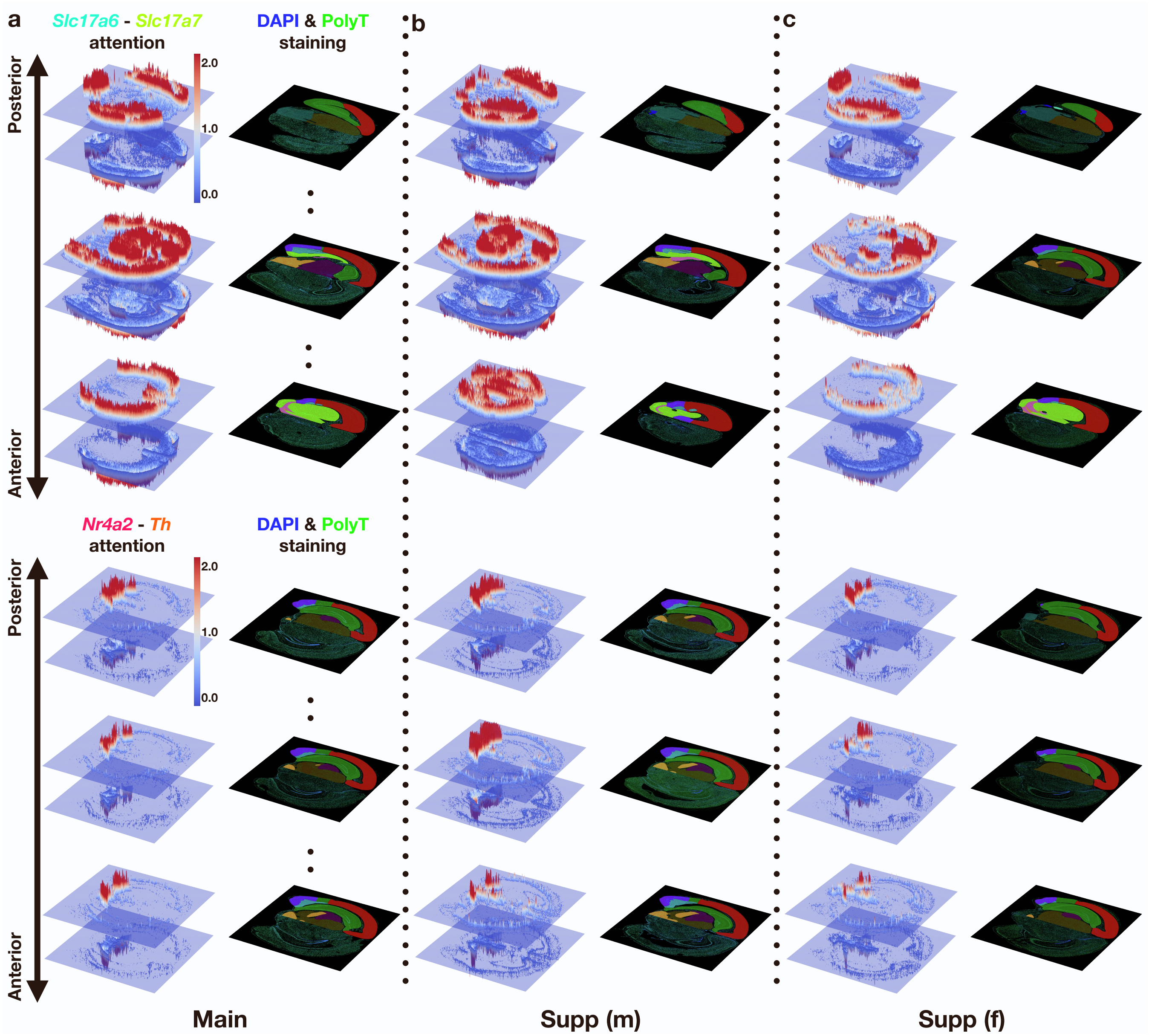

Figure 4: **The *gene-gene* interaction visualization of the previous-current and current-next slices along posterior to anterior direction for main and supp mouse brains**. **a**. The slice-wise visualization of *Slc17a6* and *Slc17a7* attention level (left) with regard to the DAPI- and PolyT-stained WSIs. **b**. The slice-wise visualization of *Nr4a2* and *Th* attention level (left) with regard to the DAPI- and PolyT-stained WSIs.

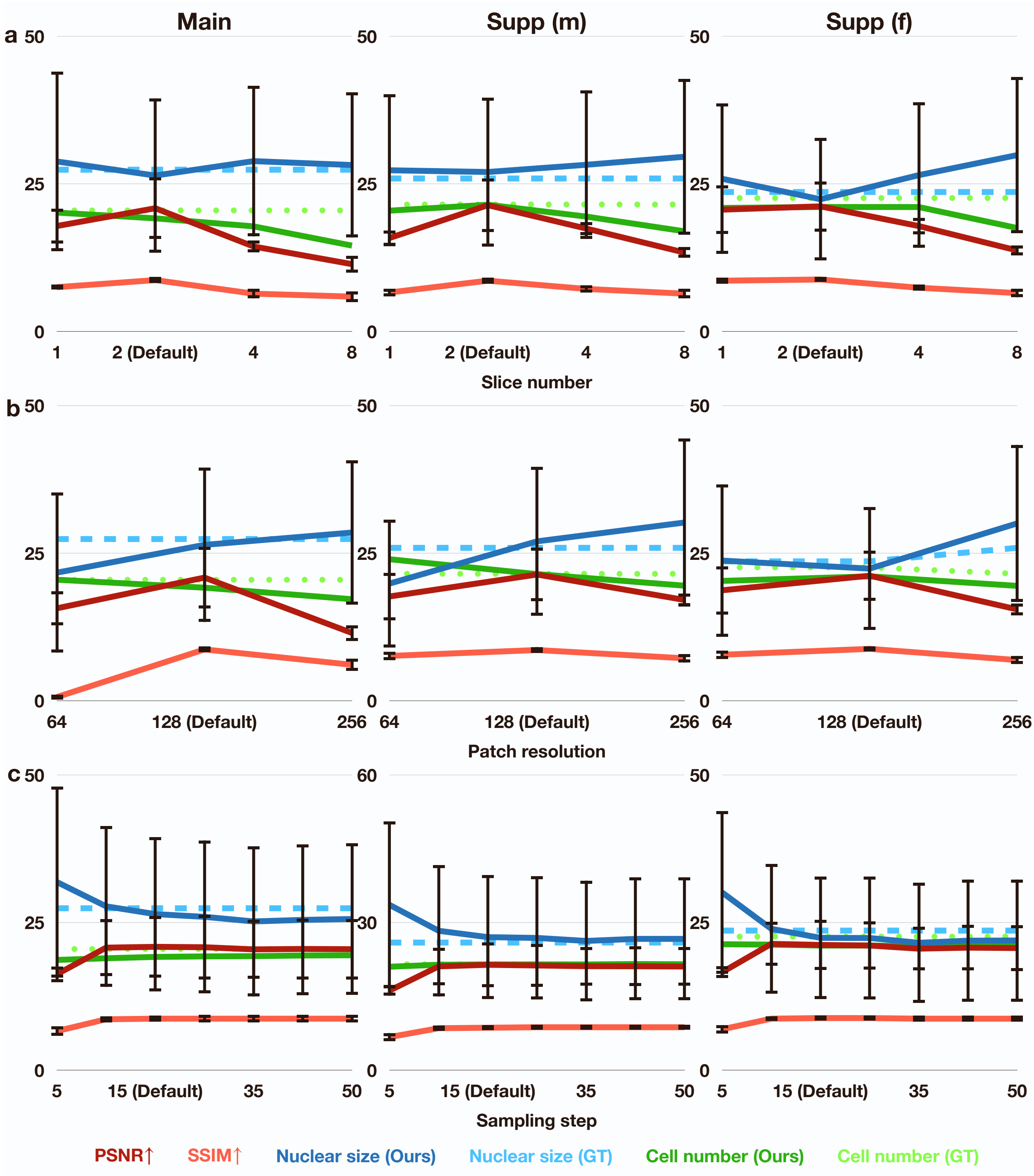

Figure 5: **The ablative results on the main and supp (m, f) mouse brains**. **a**. Quantitative performance metrics evaluated across varying slice counts. **b**. Quantitative performance metrics assessed at different spatial resolutions. **c**. Quantitative performance metrics reported for varying sampling steps. In **a**-**c**, data are presented as mean + standard deviation.
